# A Symphony of Challenges: Multi-Drug Resistant Tuberculosis, Pleurocutaneous Fistula, and Empyema Necessitans in Concert

**DOI:** 10.7759/cureus.49485

**Published:** 2023-11-27

**Authors:** Kamran Ahmad, Adeel Khan, Amna Yaseen, Mahnosh Saleh, Yasir Ali, Musa Kakakhel, Misbah Ehsan

**Affiliations:** 1 Internal Medicine, Hayatabad Medical Complex Peshawar, Peshawar, PAK; 2 Internal Medicine, Khyber Medical College, Peshawar, PAK; 3 Internal Medicine, Lady Reading Hospital Peshawar, Peshawar, PAK; 4 Medicine, Hayatabad Medical Complex Peshawar, Peshawar, PAK; 5 Internal Medicine, Ayub Teaching Hospital, Abbottabad, PAK; 6 Hematology, Khyber Girls Medical College, Peshawar, PAK; 7 Hematology, Hayatabad Medical Complex Peshawar, Peshawar, PAK

**Keywords:** uncommon clinical encounter, rare case report, tuberculosis complications, pleural infections, case report, rare presentation, tuberculous empyema necessitans, pleurocutaneous fistula, multi-drug resistant tuberculosis

## Abstract

This case report details the clinical course of a 37-year-old male with multi-drug-resistant tuberculosis (MDR-TB) who initially presented with respiratory symptoms. Following a month of anti-TB therapy, the patient developed a painful chest swelling, diagnosed as empyema necessitans, with a subsequent spontaneous rupture leading to a pleurocutaneous fistula. Despite recommendations for surgery, the patient opted for active surveillance. The follow-up revealed symptom improvement. This case underscores the unique challenges of managing rare complications of MDR-TB, particularly when patients decline surgical interventions. The observed symptom improvement, despite the absence of surgery, illuminates the intricate decision-making process and alternative management strategies involved in addressing such complications, highlighting the complexities inherent in MDR-TB care.

## Introduction

*Mycobacterium tuberculosis *causes tuberculosis. It is a disease affecting all organs, primarily the upper lung lobes. A pleurocutaneous fistula is a pathological connection between the pleural space and the subcutaneous tissues. It may arise due to an infectious condition, a tumor, foreign body inhalation, or medical interventions. They often occur in immunocompromised individuals with pyogenic infections, much less in immunocompetent patients with tuberculosis. Clinically, the onset is insidious, marked by a soft swelling with pus coming through a fistula. There may be a low-grade fever or no fever at all [1]. Diagnosis is usually made based on imaging studies such as CT scans, MRIs, transcutaneous ultrasounds, or fistulograms [2,3]. Prompt surgery and anti-tuberculous therapy are the mainstays for the management of pleuro-cutaneous fistulas [4].

## Case presentation

**Figure 1:**
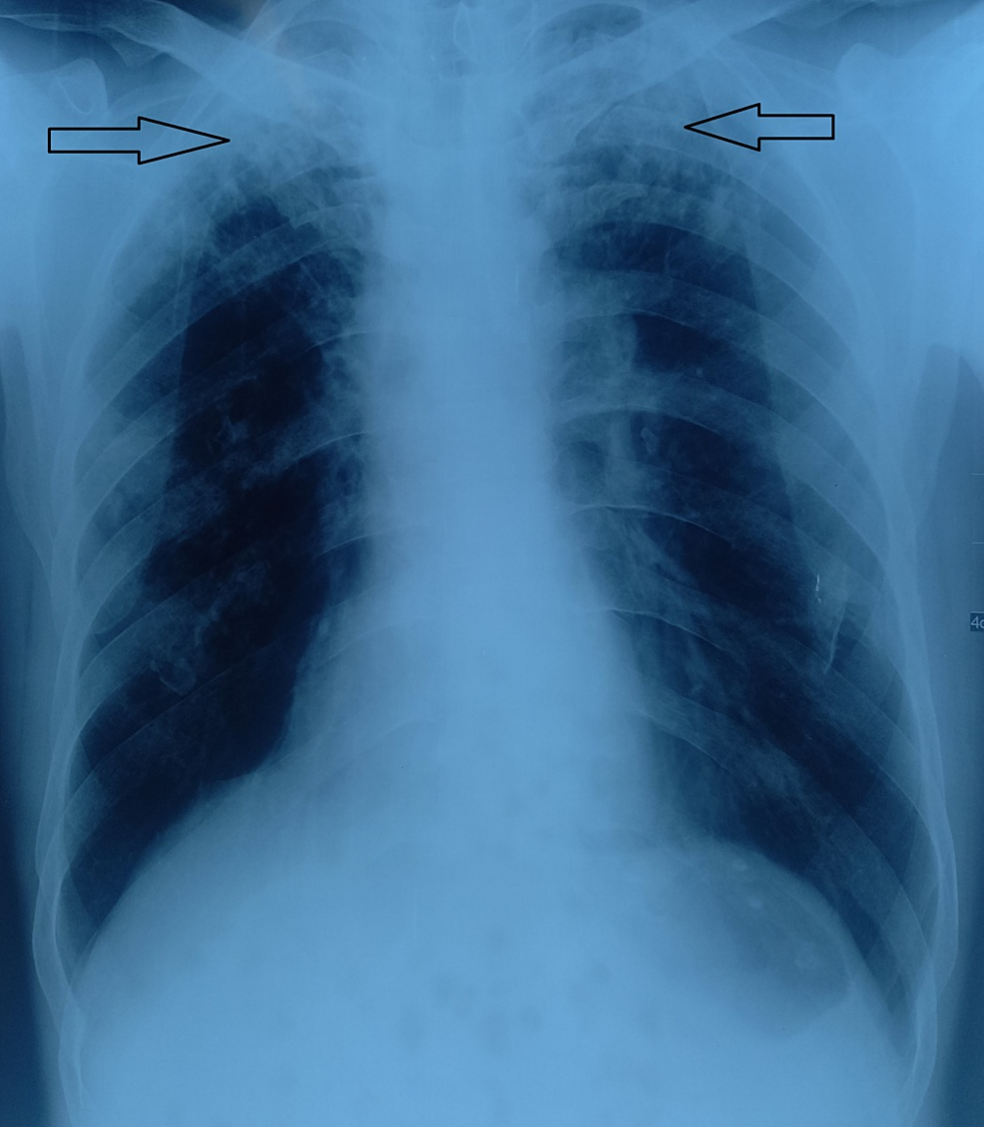
Chest X-ray showing bilateral upper lobe consolidation

A 37-year-old gentleman of South Asian descent presented to a tertiary care hospital with complaints of productive cough, malaise, fever, and night sweats for the last three months. His history was consistent with a documented weight loss of 7kg over an estimated one month. Social history revealed risk factors for active chronic smoking in the 15-pack year. Objectively, on examination, the patient was active, alert, and oriented. The patient was vitally stable with a pulse of 90, blood pressure of 130/90, and SPO2 of 95% on room air. Chest examination suggested increased breath sounds on both sides with bilateral upper zone coarse crepitations and mild wheezes on the right middle zone. His detailed workup to identify the underlying cause was done and was consistent with baseline investigations, CXR, sputum AFB, and Gene Xpert. CBC showed lymphocytic leukocytosis, and sputum AFB/Gene Xpert showed resistance to rifampicin, confirming a diagnosis of multi-drug-resistant tuberculosis. Moreover, the chest X-ray was consistent with bilateral upper lobe consolidation, as shown below in Figure 1.

**Figure 2:**
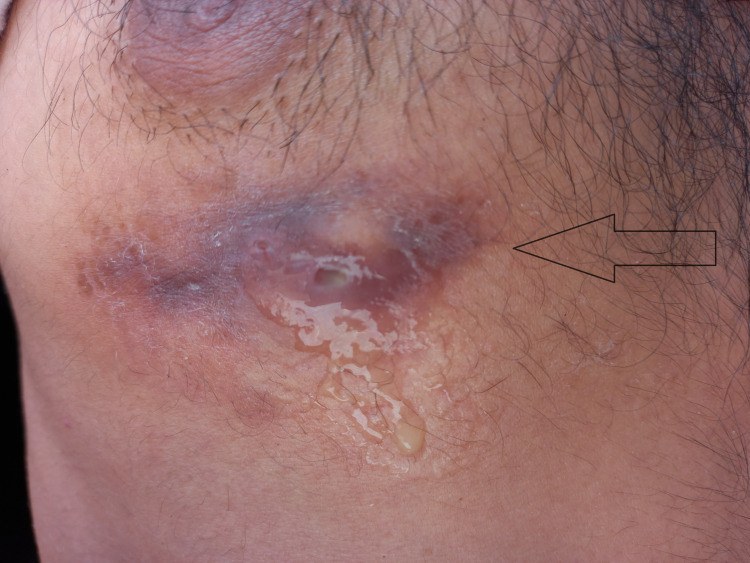
Anterior chest wall showing ruptured swelling with oozing frank pus

The patient was started on a multidrug-resistant TB regimen inclusive of Pyrazinamide 1600 mg per day, Ethambutol 400 mg, Isoniazid 300 mg, Vitamin B6 pyridoxine 50mg, Clofazimine 100mg, Bedaquillione 100 mg, and Ethionamide 50mg. One month after starting medications with due care and proper compliance, he noticed painful swelling on the right side of the chest just below the nipple associated with cough, dyspnea, and pleuritic chest pain, responsive to the use of NSAIDs. The examination was consistent with anterior chest wall swelling below the right nipple that was non-erythematous and non-tender on palpation. The percussion of the chest was dull. There was reduced air entry and breath sounds, with added right-sided basal crepitations on auscultation. An ultrasound revealed a pleural effusion. Subsequently, a diagnostic pleural tap was performed that was consistent with an exudative pattern in accordance with the Lights criterion, suggesting empyema. The Gene Xpert test yielded a positive result for tuberculosis, indicating resistance to rifampicin, consistent with multi-drug-resistant tuberculosis (MDR-TB). However, the culture of the pleural sample did not show the growth of any organisms. The patient was observed and followed for a period of two weeks and showed spontaneous rupture of swelling with frank pus ooze that was followed by clear serious discharge, as shown in Figure 2.

**Figure 3:**
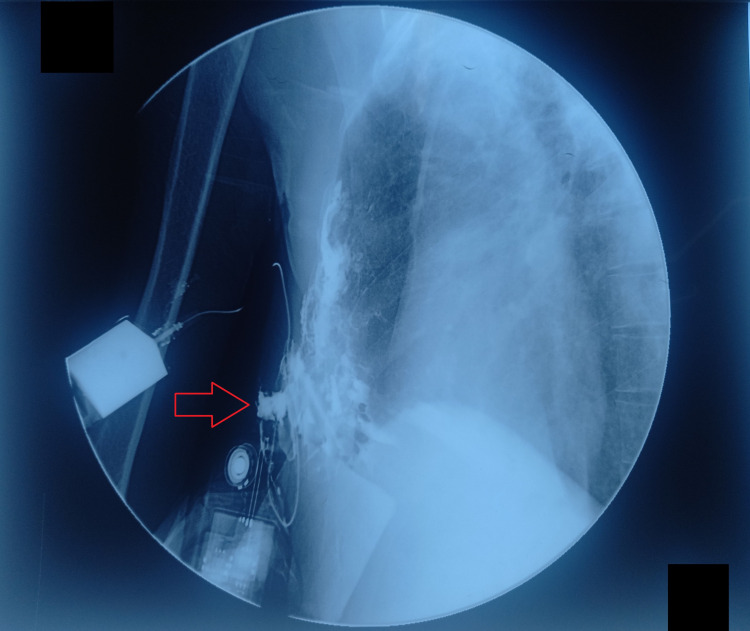
Sinogram of the chest showing the passage of contrast through an irregular complex tract dispersing into anterior pleural space, suggestive of a pleurocutaneous fistula

**Figure 4:**
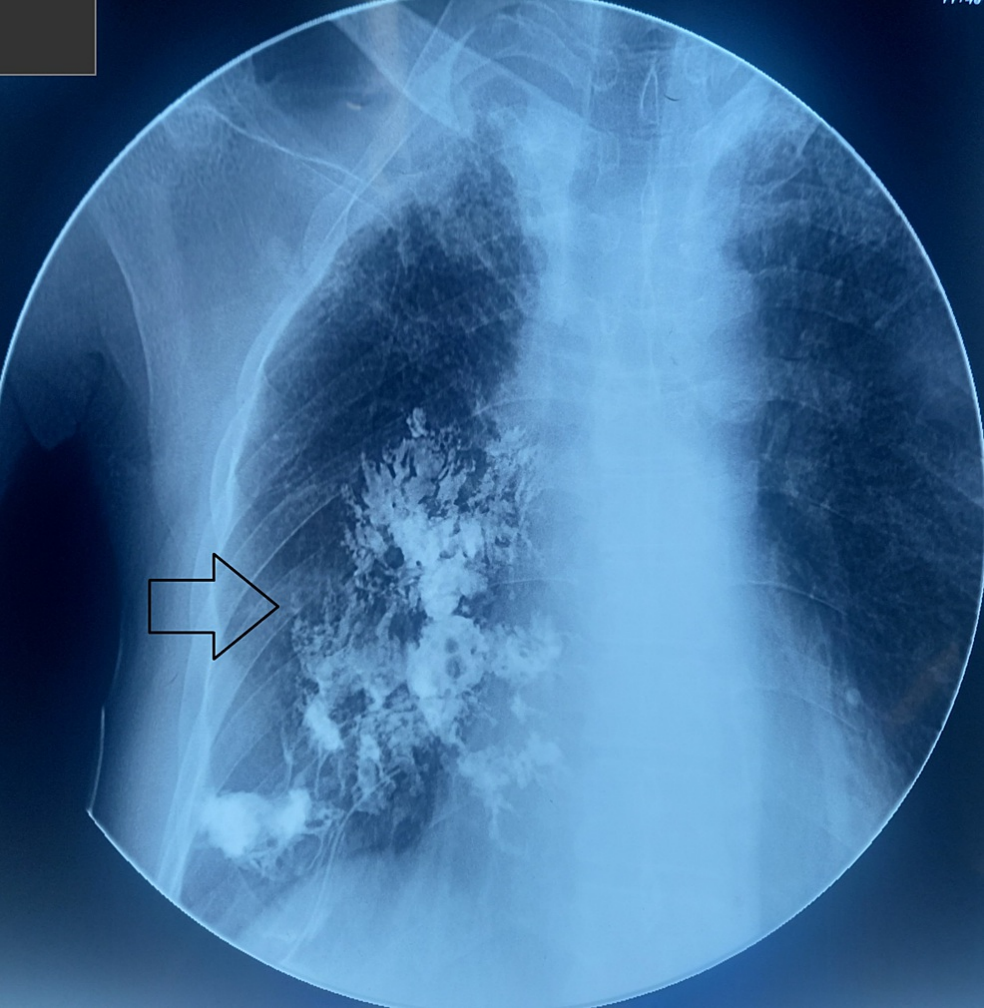
Chest sinogram showing spillage of dye into surrounding tissues, suggestive of EMPYEMA NECESSITANS

An ultrasound-guided biopsy of the lesion was taken via 18G semiautomatic tru-cut biopsy needles with three cores of biopsy, and samples were sent for histopathology. Testing came out to be negative for AFB, and cultures were negative for other potential causative microorganisms. A CT scan of the chest suggested right-sided anterior chest wall soft tissue thickening with involvement of the pleura and intrathoracic extension, hinting towards empyema necessitans. The patient was discharged home for outpatient treatment of an oral empirical antibiotic (amoxicillin + clavulanate) of 1 gram for 10 days. On follow-up after 10 days, a sinusogram was performed using a butterfly cannula with 50 ml of contrast dye into the opening of the anterior chest wall, as shown in Figures 3, 4. The sinogram confirmed the diagnosis of a pleurocutaneous fistula.

**Figure 5:**
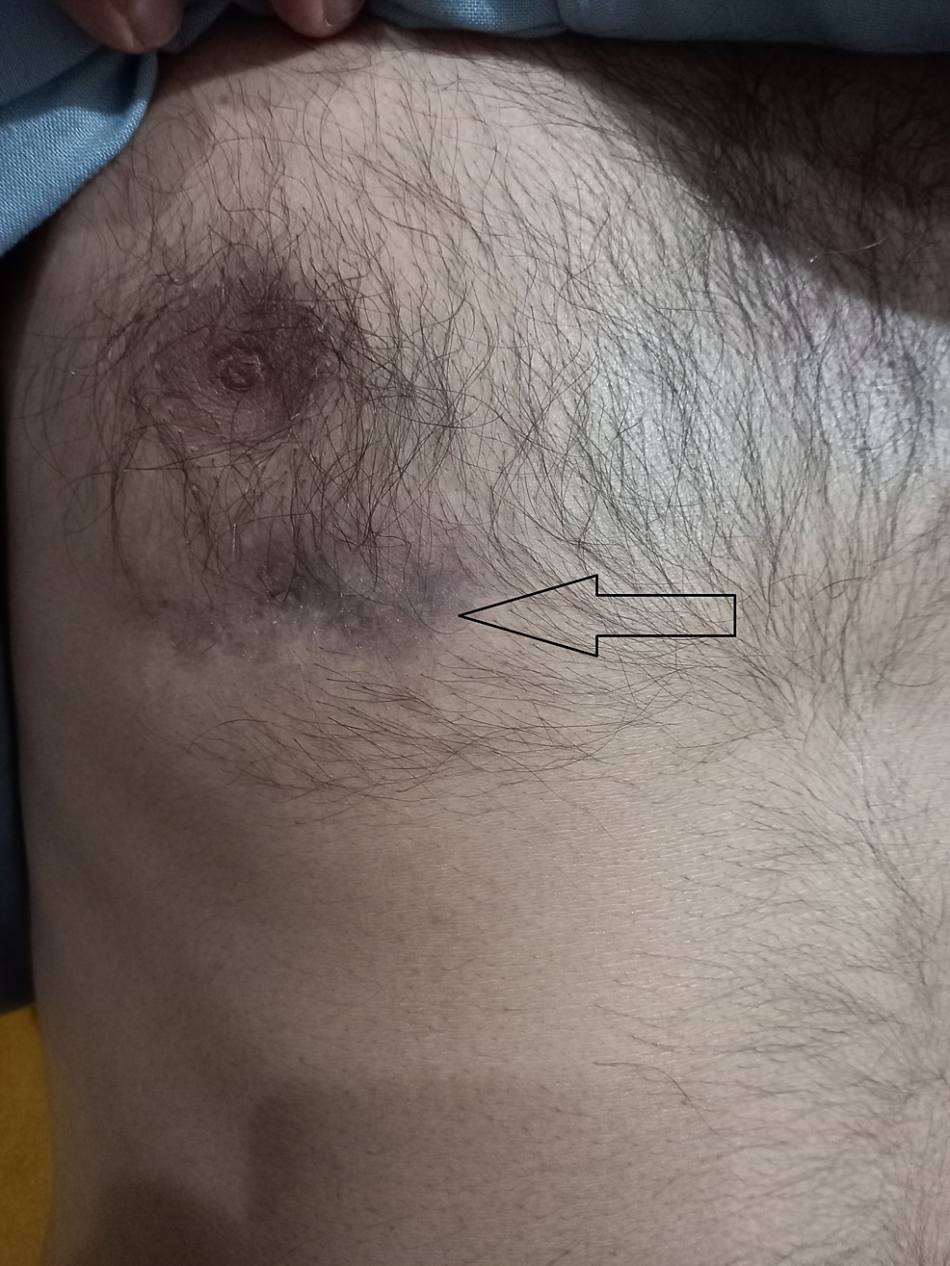
Anterior chest wall showing healed previous fistula

The patient was counseled for surgery, but he refused to go for surgical interventions despite multiple attempts at counseling and was put on active surveillance. On follow-up, there was symptomatic improvement in the patient's cough, fever, body aches, and fatigue. Drainage from the fistula had become minimal, and no active complaints were reported. As the culture report for other potential microorganisms causing similar symptoms was negative, it was more likely this was a good response to anti-tubercular therapy. A follow-up picture of the fistula can be seen below in Figure 5.

## Discussion

Pleurocutaneous fistula is a persistent communication between the pleura and the subcutaneous tissue and can be secondary to multiple causes such as an immunocompromised state, neoplasm, empyema, foreign body, thoracic surgery, or chest drain insertion [4]. The development of a percutaneous fistula has been reported following the insertion of a chest drain for tuberculous pleural effusion drainage, which paved the path for the fistula to develop. Although it is very rare, in the order of 0.1% to 1% of all cases [3]. Immunocompromised patients suffering from HIV with concomitant Hepatitis B and Hepatitis C infection diagnosed with tuberculosis developed a pleurocutaneous fistula as a result of CD4+ T cell depletion and reduction of antigen-specific cytokine responses, resulting in uncontrolled *Mycobacterium* replication [5]. This case is distinctive as there was no previous history of tuberculosis, the patient's immunocompetent state, and the absence of concomitant infections.

Thoracic interventions such as the video-assisted thoracic surgery (VATS) procedure for lung cancer or tube thoracostomy (chest tube placement) for post-esophagectomy drainage are known causes of developing pleurocutaneous fistulas [2,6]. Similarly, patients diagnosed with primary pulmonary tuberculosis started on anti-tubercular therapy, underwent chest tube placement, and also developed a pleurocutaneous fistula [4,7]. There are a number of reported causes for developing a pleurocutaneous fistula, some of which are discussed above. This case is also unique due to his young age, lack of surgical intervention, negative chest wall abnormalities, the spontaneous development of a pleurocutaneous fistula, and prompt treatment with a sensitive antitubercular regimen.

## Conclusions

In conclusion, the rare occurrence of a pleurocutaneous fistula in primary tuberculosis underscores the importance of considering this presentation in the differential diagnosis of chest wall swellings, even in the absence of predisposing conditions. Early recognition is crucial, given its potential to mimic benign lesions.

In this case, the pleural effusion was managed with standard anti-tubercular therapy, aligning with established guidelines. Surgical intervention, though recommended, was not pursued as the patient opted for active surveillance. This decision emphasizes the personalized nature of tuberculosis care. Overall, our case highlights the complexity of managing tuberculosis-associated complications and the need for a tailored approach considering both medical and surgical options.
